# Investigating the Role of Fat Mass and Obesity-Associated (FTO) Single Nucleotide Polymorphisms and Methylation in Breast Cancer

**DOI:** 10.7759/cureus.62851

**Published:** 2024-06-21

**Authors:** Marta Elena Hernández-Caballero, Jose Alfredo Sierra-Ramírez, Marlene De la Peña-Gutierrez, Fabián Galindo-Ramirez

**Affiliations:** 1 Biomedicine, Facultad de Medicina, Benemérita Universidad Autónoma de Puebla, Puebla, MEX; 2 Postgraduate Studies and Research Section, Escuela Superior de Medicina, Instituto Politécnico Nacional, Mexico City, MEX; 3 Secretariat of the Navy, Centro Médico Naval, Mexico City, MEX; 4 Meritorious Autonomous University of Puebla, Instituto de Fisiologia, Puebla, MEX

**Keywords:** rs9930506, survival, breast cancer, methylation, fto

## Abstract

Background

Fat mass and obesity-associated (FTO) protein is an mRNA demethylase enzyme essential for active genome regulation. *The FTO* gene codes for a protein that is part of the methylosome complex and has a regulatory role in cancer development. Some studies have shown a relationship between *FTO* and cancer, where single nucleotide polymorphisms (SNPs) may have some impact on cancer risk. The present study aimed to evaluate the risk of *FTO* polymorphisms rs9939609, rs1477196, and rs9930506; analyze the methylation status of *FTO* promoters among Mexican women with breast cancer (BC); and investigate by in silico analysis the methylation status in the region near these polymorphisms.

Methods

A total of 157 BC patients and 137 healthy controls were genotyped for rs9939609, rs1477196, and rs9930506 *FTO *polymorphisms by TaqMan SNP Genotyping Assays. Promoter methylation was analyzed by sodium bisulfite and methylation-specific polymerase chain reaction (MSP) for 78 tissue samples. An in silico analysis using The Cancer Genome Atlas Program (TCGA) database was employed to investigate the methylation state in promoter and near polymorphism locations and its relation to survival.

Results

The AG genotype of *FTO* rs9930506 was associated with BC protection (P= 0.0025; adjusted OR, 0.27; 95% CI: 0.10-0.70). rs9939609 and rs1477196, according to the results of the present study, had no relation to BC. Promoter methylation status assays by MSP revealed no changes in methylation in BC or healthy tissues. Trying to know more about the methylation in promoters and near polymorphisms’ relation to survival, we performed an in silico analysis. Bioinformatics analysis showed a correlation between poor survival and methylation near polymorphisms but not with methylation in the promoter region.

Conclusions

The AG genotype rs9930506 has a protective function against BC. Whereas high methylation near polymorphisms was related to lower survival, the hypomethylated promoter region does not impact survival.

## Introduction

Fat mass and obesity-associated (FTO) is a ubiquitous DNA repair enzyme homolog of the AlkB family of Fe(II)/α-Ketoglutarate-dependent dioxygenases. FTO catalyzes the demethylation of RNA and DNA substrates, but recent studies point toward modifications in mRNA as its most relevant substrate with genome-wide effects [1]. In early studies of FTO function that focused on the relationship of single nucleotide polymorphisms (SNPs) with cancer, variable results regarding the association of FTO SNPs with BC have been reported in populations of different ethnic origins [2-4]. FTO variants have a strong relationship with changes in BMI and body weight [3]. Different studies have reported an oncogenic role for FTO, which can act as an oncogene when upregulated or as a tumor suppressor when downregulated [5]. DNA methylation is another mechanism of transcriptional regulation; changes in promoter methylation of genes alter their expression patterns in an epigenetic manner, which is usually attributed to loss of gene function. The addition of a methyl group modifies a cytosine base located 5’ to guanosine in a CpG dinucleotide [6]. CpG islands are predominantly nonmethylated and present in 70% of all mammalian promoters [7]. Hypermethylation of promoters is the most common epigenetic change in tumors [8]. Similar to DNA methylation, RNA can be modified by methylation continuously and reversibly. Gerken et al. [9] demonstrated that the FTO protein is an mRNA demethylase with an essential activity for genome function. The recent discovery of FTO as an active posttranscriptional RNA modifier has provided additional insight into the regulatory role of genes in cancer. FTO belongs to one of the two families of enzymes responsible for maintaining the balance of N6-methyladenosine (m6A) modification: methyltransferases and demethylases [10].

Despite great advances in BC treatment, its appearance, development, and cellular interactions are extraordinarily complex and cannot be easily solved. Owing to the development of gene sequencing technology, it has become possible to analyze mutational signatures in cancer genomes and capture specific clinically relevant biomarkers [11]. This study aims to evaluate the association between rs9939609, rs1477196, and rs9930506 FTO polymorphisms and BC, as well as to analyze the methylation status of the FTO promoter and investigate by in silico analysis the methylation status in the region near these polymorphisms in BC patients.

## Materials and methods

Participants

The study included 157 patients with BC and 137 controls without BC. BC patients were recruited at the Centro Médico Nacional La Raza, Instituto Mexicano del Seguro Social in Mexico City, Mexico. Participants included women aged 32-87 years diagnosed with BC and women without BC from the general population aged 25-97 years who were invited to participate as controls.

Data collection

Epidemiological and clinical data (weight, menarche, menopause, alcohol intake, smoking, medical history, tumor stage at diagnosis) were collected using structured questionnaires and medical records (see Appendix).

Inclusion and exclusion criteria

The inclusion criteria were as follows: patients with a confirmed BC diagnosis, without prior administration of any radiotherapy or chemotherapy, and a first-degree familial history of BC. Healthy women from the general population without first-degree familial records of BC. Women who met any of the following criteria were excluded: prior first-degree familial history of BC, patients with completed cancer treatment by radiotherapy or chemotherapy, and patients who did not want to participate. In the case of healthy women, those with a history of BC in the family or who did not wish to participate in the study were excluded.

Ethical approval

The study protocol was approved by the institutional research committee from Centro Médico Nacional La Raza, Instituto Mexicano del Seguro Social in Mexico City, Mexico (671/12), and each participant gave written informed consent. This study was conducted under the Declaration of Helsinki.

DNA extraction

Three milliliters of peripheral blood were obtained in EDTA vacuum tubes from patients and controls for genotyping analysis of the three studied polymorphisms. For the methylation assay, tissue samples were obtained prior to the administration of any radiotherapy or chemotherapy from 78 of those BC patients. Genomic DNA was extracted using the QIAamp DNA Blood Mini Kit according to the manufacturer’s protocol (Qiagen, Valencia, USA). Once DNA was obtained, it was stored at -40 °C. These polymorphisms, rs9939609, rs1477196, and rs9930506, were analyzed. All TaqMan SNP probes were purchased from ThermoFisher Scientific (ThermoFisher Scientific, San Francisco, USA); their assay IDs are C__30090620_10, C___2031262_10, and C__29819994_10, respectively. Genotyping assay was performed using TaqMan Genotyping Master Mix (ThermoFisher Scientific) following the manufacturer’s instructions. All polymerase chain reactions (PCRs) were performed in a volume of 10 μL with 30 ng of genomic DNA. We included two negative controls (water) on each plate. Assays were measured using an Applied Biosystem StepOne 7500 Real-Time PCR System (ThermoFisher); genotypes were determined using the manufacturer’s software.

Promoter methylation analysis by methylation-specific PCR (MSP)

The promoter methylation status of the FTO gene was examined in 78 sporadic BC tumors and 18 non-tumor adjacent tissues. To analyze promoter methylation, DNA isolated from tissues was bisulfite modified using an EpiTect Bisulfite Kit (Qiagen) in accordance with the manufacturer’s protocol as previously described [12]. A CpG island from the promoter region was located using the Eukaryotic Promoter Database tool [13]. MSP primer pairs were designed using Methprimer software [14] to detect bisulfate-induced changes affecting unmethylated (U) and methylated (M) alleles. The primer sequences used are shown in Table 1. PCR for bisulfate-converted DNA was performed using an EpiTect MSP Kit (Qiagen). Twenty nanograms of DNA, 10 mM of each primer, and 2X Master Mix MSP in a final reaction volume of 10 mL were prepared. Cycle conditions for methylation detection are described in Table 1. Each PCR assay included a methylation control, an unmethylated control, and genomic DNA (EpiTect PCR Control DNA Set, Qiagen). The PCR products were resolved by 3.5% agarose gel electrophoresis for 80 minutes under a constant voltage of 100 V in a CScientific electrophoresis cell using 1xTBE buffer. The gels were visualized on a UV gel transilluminator UVP (Analytik Jena, USA).

**Table 1:** FTO primers for MSP MSP, methylation-specific PCR; PCR, polymerase chain reaction

Primers	Sequences (5’-3’)	PCR product sizes	PCR conditions
Methylated		103 bp	95 °C 10 minutes, 40 cycles at 95 °C 15 seconds, 59 °C 30 seconds, 72 °C 30 seconds followed by 72 °C 7 minutes
M-F	TCCTACATATAATTCTCTCAACGTC		
M-R	TTTTTGGATTTTAGTTTGTTTGTTC		
Unmethylated		102 bp	95 °C 10 minutes, 35 cycles at 94 °C 15 seconds, 55 °C 30 seconds, 72 °C 20 seconds followed by 72 °C 10 minutes
U-F	TCCTACATATAATTCTCTCAACATC		
U-R	TTTTTGGATTTTAGTTTGTTTGTTTG		

Bioinformatic assay

Since the MSP technique that we used did not allow us to analyze individual CpG sites, we explored the MEXPRESS tool (https://mexpress.be) [15]. This data was based on methylated DNA profiles of autosomal CpG sites using the Illumina Infinium HumanMethylation450 BeadChip. MEXPRESS allows us to see each probe number, its location in the gene, and obtain the methylation values (methylation levels are estimated based on the intensity of probes; the beta-value is the ratio of the methylated probe intensity to the overall intensity; the percentage of methylation is from 0% to 1% or 0% to 100%). Next, with this information, we used the MethSurv database (https://biit.cs.ut.ee/methsurv) to obtain the relation between methylation level in the CpG sites nearer the FTO gene SNPs and survival in BC [16].

Next, data mining of BC gene methylation to explore the relationship between FTO methylation and tumor type was done in 782 patients from The Cancer Genome Atlas Program (TCGA) database. We found seven probes, six in the promoter region and one in the gene body, which was the nearest to the polymorphism region (cg06054593, cg06348615, cg02859443, cg27021131, cg08467261, cg08171876, and cg01485549, respectively).

Statistical analysis

Since there were multiple variables, categorical and numerical, principal component analysis (PCA) was carried out. To optimize the PCA, the categorical variables were transformed into numerical variables by assigning them values from 0 to 9, without any hierarchy, except for polymorphisms. Given that BMI is important and has been suggested as a risk factor in cancer, to determine its relevance in this study, two PCAs were performed, one considering BMI and the other without it. In the case of any polymorphism related to cancer, to determine whether the frequency of presentation of its alleles was related to the disease and BMI, a Chi-square test was performed. The α level was set at 0.05, and the R version 4.3.1 software was used for calculations.

The genotype, allele frequency, Hardy-Weinberg equilibrium (HWE) of each FTO gene SNP, and association between genotypes and BC were evaluated by SNPStats (https://www.snpstats.net/start.htm), with four genetic models (co-dominant, dominant, recessive, and overdominant). To control confounding factors, covariates (age, BMI, alcohol intake, smoking, and menopause) were added to the genetic models for adjustment. A P-value >0.05 was considered significant.

## Results

The study group consisted of 157 patients aged between 32 and 87 years (56.4 ± 11.39 years), and 137 controls aged from 25 to 97 years (51.4 ± 12.83 years). The general characteristics of both groups are described in Table 2.

**Table 2:** General characteristics of controls and patients ^a ^Weight is grouped according to BMI (ranges according to the Mexican Institute of Social Security) ^b ^n = 137 ^c ^n = 157 ** denotes significant P-value

Factor	Controls^b^, n (%)	Cases^c^, n (%)	X^2^	P-value
BMI (kg/m^2^)^a^			102.21	<0.001**
Underweight (<18.5)	5 (3.6)	0 (0)		
Normal weight (18.5-24.9)	95 (69.3)	30 (19.1)		
Overweight (25-30>) and obesity	34 (24.8)	127 (80.8)		
Smoke			33.18	<0.001**
No	115 (83.9)	99 (63)		
Yes	22 (16)	25 (15.9)		
Unknown	0 (0)	33 (21)		
Menarche			3.47	0.06
12≤	54 (39.4)	80 (50.9)		
≥12	83 (60.5)	77 (49)		
Menopause			86.07	<0.001**
No	94 (68.6)	24 (15.2)		
Yes	43 (31.3)	125 (79.6)		
Unknown	0 (0)	8 (5)		
Alcohol intake			32.55	<0.001**
No	122 (89.5)	112 (71.3)		
Yes	15 (10.9)	12 (7.6)		
Unknown	0 (0)	33 (21)		

After reviewing patients' medical records, we observed that most cases were diagnosed at stage II (44%) or stage III (47.4%). The most frequent tumor type was invasive ductal carcinoma (IDC, 77.7%), followed by infiltrating lobular carcinoma (ILC, 18.5%). The correlation assay revealed a significant association with cancer and the SNP rs9930506 (P = 0.41) and no significant association with cancer and the SNPs rs9939609 (P = 0.53) and rs1477196 (P = 0.78) in cases and controls. rs9930506G was higher in cases than in the control group, as shown in Table 3.

**Table 3:** Distribution of allelic and genotypic frequencies of FTO polymorphisms ^w^ wild type ^m^ mutant type * denotes significant P-value A, adenine; C, cytosine; FTO, fat mass and obesity-associated; G, guanine; T, thymine

Factor	Controls (%)	Cases (%)	X^2^	P-value
rs9939609			1.25	0.53
T^w^	225 (82)	257 (82)		
A^m^	49 (18)	57 (18)		
A/T	41 (30)	41 (26)		
A/A	4 (3)	8 (5)		
T/T	92 (67)	108 (69)		
rs1477196			0.48	0.78
A^w^	142 (52)	167 (53)		
G^m^	132 (48)	147 (47)		
A/A	38 (28)	43 (27)		
A/G	66 (48)	81 (52)		
G/G	33 (24)	33 (21)		
rs9930506			6.34	0.041*
A^w^	212 (77)	232 (74)		
G^m^	62 (23)	82 (26)		
A/A	85 (62)	99 (63)		
A/G	42 (31)	34 (22)		
G/G	10 (7)	24 (15)		

rs9930506 AG was significant under codominant and overdominant models; it was a protective factor (P = 0.0025; adjusted OR: 0.27; 95% CI: 0.10-0.70, Table 4). The HWE test revealed that the genotype distributions of rs9939609 (P = 0.33) and rs1477196 (P = 1) were consistent with HWE, but rs9930506 deviated from HWE in patients (P = 0.0001).

**Table 4:** FTO rs9930506 association with BC (adjusted by age, smoke, alcohol intake, and menopause) * denotes a significant P-value A, adenine; BC, breast cancer; C, cytosine; FTO, fat mass and obesity-associated; G, guanine; T, thymine

Model	Genotype	Controls (%)	Cases (%)	OR (95% CI)	P-value
Codominant	A/A	85 (62)	99 (63.1)	1	0.0025*
	A/G	42 (30.7)	34 (21.7)	0.27 (0.10-0.70)	
	G/G	10 (7.3)	24 (15.3)	2.11 (0.72-6.15)	
Dominant	A/A	85 (62)	99 (63.1)	1	0.2
	A/G-G/G	52 (38)	58 (36.9)	0.62 (0.30-1.29)	
Recessive	A/A-A/G	127 (92.7)	133 (84.7)	1	0.047
	G/G	10 (7.3)	24 (15.3)	2.84 (1.00-8.08)	
Overdominant	A/A-G/G	95 (69.3)	123 (78.3)	1	0.0015*
	A/G	42 (30.7)	34 (21.7)	0.24 (0.09-0.61)	

**Figure 1:**
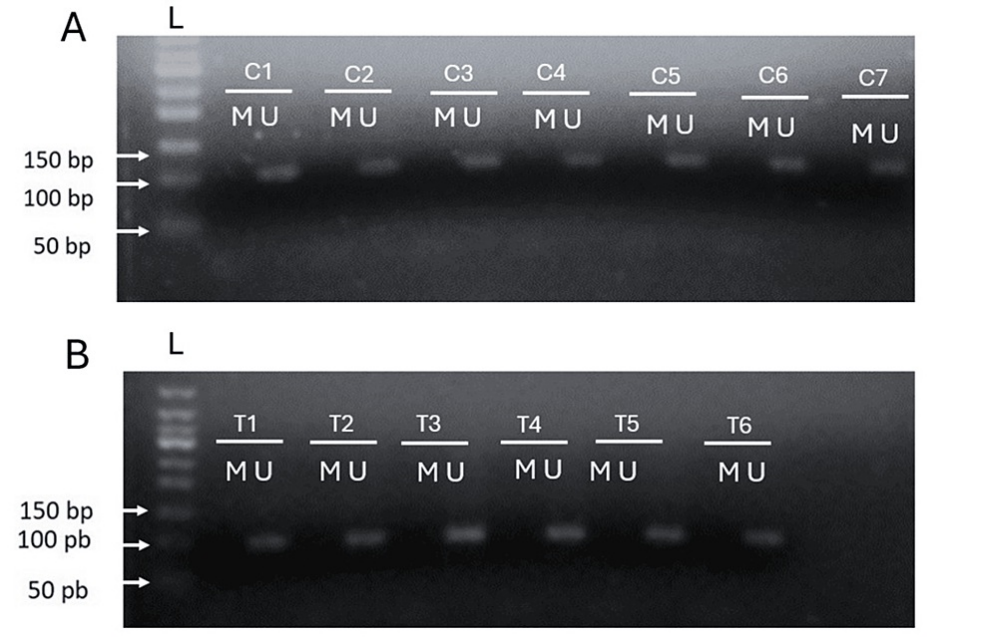
3.5% gel electrophoresis for FTO promoter methylation A representative image of MSP to FTO promoter in healthy (A) and tumor tissues (B). Each representative tissue, control, or tumor includes two wells, where U represents unmethylated amplicon and M represents methylated amplicon. C, control; FTO, fat mass and obesity-associated; L, 50 bp DNA ladder; MSP, methylation-specific polymerase chain reaction; T, tumoral

Sodium bisulfite catalyzes the conversion of cytosine to uracil in non-methylated DNA, but not in methylated DNA. The methylation assay revealed no changes in FTO promoter methylation status in BC compared with healthy tissues, and all tissues were demethylated (Figure 1).

**Figure 2:**
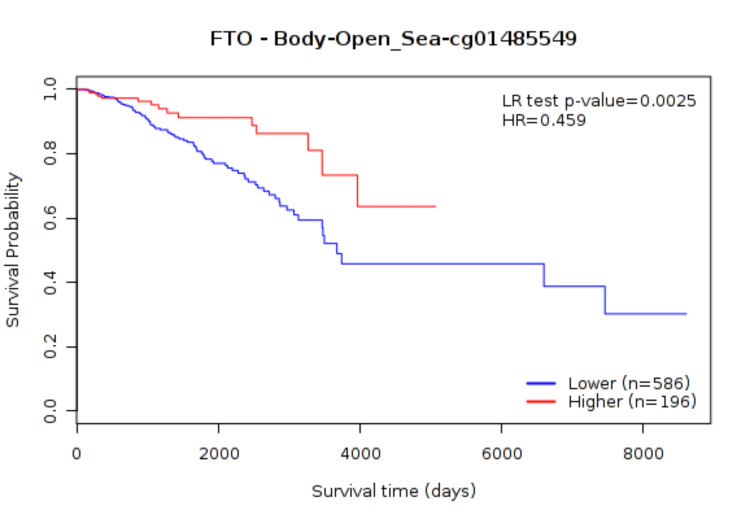
Kaplan-Meier survival analysis It is shown that patients with high methylation in cg01485549 have shorter overall survival (P = 0.0025).

We compared the probe’s status methylation and tumor type, IDC and ILC, using the U de Mann-Whitney test (data without normal distribution error). Probe cg01485549, the nearest to the polymorphism region, had the highest methylation b-value of 0.74 in IDC and 0.75 in ILC, as is observed in Table 5. The analysis in MethSurv based on the CpG methylation pattern provided us with a potential prognosis relating to high methylation with lower survival, P = 0.0025 (Figure 2).

**Table 5:** Methylation status of FTO (b-value) ^a ^mean ± SD FTO, fat mass and obesity-associated; IDC, invasive ductal carcinoma; ILC, infiltrating lobular carcinoma

Probe	Tumor type			
Promoter	n	IDC^a^	n	ILC^a^
cg06054593	574	0.037 ± 0.020	184	0.033 ± 0.008
cg06348615	574	0.048 ± 0.023	184	0.041 ± 0.012
cg02859443	574	0.050 ± 0.025	184	0.045 ± 0.018
cg27021131	574	0.043 ± 0.020	184	0.036 ± 0.011
cg08467261	574	0.016 ± 0.004	184	0.016 ± 0.003
cg08171876	574	0.038 ± 0.013	184	0.040 ± 0.012
Gene body				
cg01485549	574	0.74 ± 0.14	184	0.75 ± 0.11

## Discussion

SNPs are the most common type of genetic variation that causes individual differences in susceptibility to diseases, including cancer. SNPs are in coding and non-coding areas, such as introns. This study investigated and analyzed three intronic SNPs, the FTO polymorphisms rs9939609, rs1477196, and rs9930506, in women with and without BC. Our results showed that the GG genotype was a risk factor (OR = 2.9, 95% CI: 1.3-7.0, P = 0.006); however, after adjusting for age, BMI, smoking, alcohol intake, and menopause, this genotype was not a risk factor (OR = 2.4, 95% CI: 0.8-6.7, P = 0.09), suggesting that the association of the GG genotype with BC could be affected by other variables that could be effect modifiers or even confounders. In contrast, carriers of the AG genotype rs9930506 showed a lower risk for BC even after adjusted analysis; the AG genotype of FTO rs9930506 was more frequent in the control group compared with BC patients (P = 0.0025), supporting a protector role. A study on the relationship between rs9930506 and BC was conducted in 134 Polish patients and did not show any significant association [4]. This difference may be the result of genotype distributions: A/A 27%, A/G 48%, and G/G 25% (as shown in Table 3). Also, when we compared Mexican women with and without BC, they did not mention their control group characteristics. The other SNP, rs9939609, had a distribution that was not statistically significant between the two groups. According to the present study, rs9939609 was not associated with BC, the result is consistent with other studies. Mojaver et al. analyzed Iranian patients, finding no association between rs9939609 and an increased risk of BC (P > 0.05) [17]. In another investigation, Li et al. performed a meta-analysis with 13 studies, and their results suggest that FTO rs9939609 was not significantly associated with cancer risk (OR = 1.01, 95%CI: 0.98-1.04) [18]. Only Zeng et al. found an association of rs9939609 with BC, but in the haplotype rs9939609-rs1477196-rs1121980 (P = 0.009) [19]. The other polymorphism analyzed, rs1477196, in our study showed no significant association with BC. Previous studies have shown contradictory findings: no association with cancer risk in 199 Iranian women [17], association with BC risk in 718 women with Caucasian majority [2], or reduced BC risk in a case-control study with 1,074 Chinese women with BMI<24 kg/m2 and AA genotype [19]. These differences could be the result of differences in sample size or ethnic differences among those studies. Although previous studies have tried to find an association between FTO polymorphisms and BC, the most significant BC association is with BMI. rs9939609 variant allele A has been linked to the increase in BMI by GWAS in people with European ancestry, in whom this variant is more common [20,21], but studies on different Asian ethnicities or Hispanic populations have reported the same result. A meta-analysis performed by Zhao et al. identified a strong association between FTO SNPs and obesity risk in the Chinese population (OR 1.30; 95% CI: 1.19-1.42, P < 0.0001) [22]. Park and Choi showed that FTO rs9939609 is associated with obesity risk in Korean women [23]. A study conducted by Perez-Luque et al. on the Mexican population found that rs9939609, rs9930506, and rs1421085 are key to gaining weight after bariatric surgery [24]. The work of Martinez-López et al. in the Mexican population also supports the role of rs9939609 on class III obesity [25]. It is noteworthy that the obesity risk allele A is not the most frequent one among Mexicans; instead, in our population, rs9939609 has been previously associated with obesity class III [26]. For the SNP rs9930506, in a study on 2,314 unrelated Mexican adult subjects, rs9930506G has been associated with a significant increase in BMI in a gender-dependent manner (OR = 4.4 in women vs. men) [27]. Recently, Gholami, through the identification of common variants associated with BC, obesity, and diabetes, found that the FTO gene is a major gene shared between these three diseases, with rs9930506 as one of those variants involved [28]. Our results showed that rs9930506 GG was associated with BC (P = 0.041), and genotype AG was a protective factor (P = 0.0025).

The state of DNA methylation of CpG in promoters and gene bodies is associated with transcriptional activity in genes, and epigenetic changes have been related to illness. Our study analyzed the methylation status of the FTO promoter by MSP in BC patient tissues. This technique is based on the use of primers that bind to methylated sequences and primers that bind to unmethylated sequences. We did not find significant differences in FTO promoter methylation between BC and healthy adjacent tissues. Electrophoresis revealed no difference, showing that both tissues were demethylated. FTO promoter methylation has been analyzed in pathologies such as metabolic syndrome, where increased levels of DNA methylation were found compared to participants without any metabolic conditions [29]. In one study that analyzed a CpG site in the first intron of FTO, the authors observed small but significant hypomethylation (P = 0.000021) upstream rs1121980 FTO polymorphism in patients with type 2 diabetes mellitus (T2DM) relative to controls [30]. The use of databases has allowed us to elucidate the vital roles of genes in tumorigenesis. So, we researched the DNA methylation levels of FTO and the predictive value of CpG islands in the promoter and near the polymorphism in the TCGA database with the MethSurv tool. We found that FTO hypermethylation near the polymorphism region was associated with poorer survival (P = 0.0025).

The study has limitations; the deviation from HWE in cases (but not in controls) may be due to the possible genetic association between FTO SNPs, obesity, and BC. However, this research has the advantage of being conducted in a Mexican population, where other studies have analyzed FTO SNPs, but with a focus on obesity and T2DM rather than cancer. Another strength of this study is the rigorous scientific retrieval strategy used to identify and review relevant literature.

## Conclusions

Our analysis shows that FTO demethylase presents an association between the rs9930506 FTO alleles with BC (GG) and without BC (AG). On the other side, hypermethylation in CpG near the polymorphism region is associated with poorer survival, contrasting with the hypomethylated promoter region, which was not related to survival. These results suggest that FTO SNP rs9930506 may have a role in BC development; this role is supported by the close genetic association between this cancer and obesity, pathologies in which the FTO gene is involved.

## Appendices

Data collection sheet.                                                           Number:_____

Study of the polymorphism rs1477196, rs9930506, and rs9939609 of the gen FTO in women diagnosed with breast cancer in Mexican population.

Hospital:                                       Date:___/___/____   Afiliation number___________

Identifier:______________________________________ Age:_____________

Birth place : ___________. City/State ______________

Mexican grandparents:  YES  ___________       NO ____________

Scholarship: Illiterate________ Primary school______   High school_______

                   Degree_______   Master’s degree______   Doctorate _______

Occupation:__________________

History of hypothyroidism: Yes ____   No  _____

History of Mellitus diabetes: Yes _____ No _____

History of Adrenal disease: Yes _____ No_____

History of Family cancer: Yes _____ No _____

Which ones? :____________________________________________________

Actual weight :___________ Height:_______

Do you smoke?___

How much alcohol do you drink in a week (glasses): ______

Total Pregnancies____ Deliveries ____. Abortions:____ Cesarean sections____ Breastfeeding: Yes___ No____

Age of menarche:_____

Do you use hormonal contraceptives? :___________

Menopausia (Age):______
